# Human Skin RNases Offer Dual Protection against Invading Bacteria

**DOI:** 10.3389/fmicb.2017.00624

**Published:** 2017-04-11

**Authors:** Bin Wang

**Affiliations:** Department of Chemistry, Marshall University, Huntington, WV, USA

**Keywords:** human skin RNases, Rho-dependent transcription terminators, Rho utilization site, RNase A superfamily, desquamin

To control the growth of microorganisms on its surface, human skin produces and releases certain peptides and proteins, including cathelicidin LL-37 and β-defensins. The ribonuclease A (RNase A) superfamily is an important class in this armament (Koczera et al., 2016). This superfamily consists of 13 genes located on chromosome 14; eight of the 13 genes encode proteins (RNases 1–8) that are catalytically active on RNA substrates (Abtin et al., 2009; Simanski et al., 2012; Prats-Ejarque et al., 2016; Pulido et al., 2016).

Among the eight catalytically active RNases in the RNase A superfamily, only RNases 1, 4, 5, and 7 are expressed in human keratinocytes (Abtin et al., 2009; Simanski et al., 2012). The expression level of RNase 7 is the highest among these four RNases, and only RNases 5 and 7 exhibit antibacterial activity *in vitro*. The bactericidal action of RNase 7 is based on its binding to and destabilization of the bacterial membrane by way of an electrostatically driven protein-membrane association followed by the formation of transient “holes” on the destabilized membrane, causing the leakage/release of the inner contents (Torrent et al., 2009). RNase 7 exhibits a broad-spectrum activity against a variety of Gram-positive and Gram-negative bacteria including *Staphylococcus aureus, Enterococcus faecium, Escherichia coli, Pseudomonas aeruginosa, Klebsiella pneumonia*, and *Proteus mirabilis* (Pulido et al., 2013; Wang, 2014; Rademacher et al., 2016,2017). This bactericidal activity is independent of its ribonucleolytic activity (Simanski et al., 2012).

The antimicrobial activity of RNase 5 has been observed against the Gram-positive bacterium *Streptococcus pneumoniae* and the yeast *Candida albicans* (Hooper et al., 2003). Abtin et al. found that the antifungal activity of RNase 5 against *C. albicans* requires its ribonucleolytic activity (Abtin et al., 2009; Zasloff, 2009). It is not currently known whether its antibacterial activity against *S. pneumoniae* requires the same prerequisite/co-requisite.

In addition to their antipathogenic activity, the ribonucleolytic activity of RNases on human skin has been investigated. Steve Pascolo's research group discovered that naturally-occurring RNase activity on the surface of human skin specifically targets cytosine (C) residues (Probst et al., 2006). They tested synthetic single-stranded RNA homopolymers 18 nucleotides in length, and determined that while poly(A), poly(G), and poly(U) oligonucleotides remain intact in the presence of human skin RNases, poly(C) oligonucleotides are degraded (Probst et al., 2006).

Other human skin proteins also display ribonuclease activity. Miriam Brysk's research group reported that desquamins, glycoproteins not belonging to the RNase A superfamily, have demonstrated both RNase and protease activity (Selvanayagam et al., 1998). Desquamins are expressed in the transition zone between the granular layer and the stratum corneum. Functioning as RNases, desquamins have been found to degrade only poly(C) RNA; homopolymers of A, U, and G are not affected (Selvanayagam et al., 1998).

The author experimentally investigates RNA structure, and has found that even with the strictest precautions during experimental procedures, RNA transcripts are occasionally cut at C residues, leading to the conclusion that human skin is the only remaining probable source of RNase contamination. Based on existing and/or predicted RNA structural information, the cleavage at C nucleotides always occurs in single-stranded portions of their RNA molecules (Wang et al., 2008).

The GC content in bacteria varies widely, from approximately 14 to 75% (Agashe and Shankar, 2014). Why do the RNases on the surface of human skin specifically cleave single-stranded C nucleotides of RNA? The author proposes that the target specificity of human skin RNases may be due to certain characteristic C-rich motif(s) widely distributed within bacterial genomes. Current knowledge suggests that an unstructured C-rich motif is widespread among bacterial RNAs: the Rho-dependent transcription terminators, which consist of single-stranded C-rich sequences, known as a Rho utilization site (*rut*), in the nascent RNA. Once bound to the *rut* site in a newly transcribed RNA, the ATPase activity of Rho is activated, driving its translocation down the RNA. Rho then catches up with the RNA polymerase; the interaction between the Rho protein and the RNA polymerase complex stimulates the dissociation of the transcriptional complex, which results in the termination of transcription. While *rut* sites are C-rich, a clear consensus sequence has not been identified (Peters et al., 2009).

Rho-dependent termination accounts for up to half of all transcription termination events in bacteria. Rho-dependent terminators are located at the 3′ ends of genes (intergenic), within the coding sequences of genes (intragenic), and even in the 5′ leader regions (to regulate transcriptional elongation of specific genes into their respective downstream coding regions) (Peters et al., 2009; Hollands et al., 2012, 2014). The author hypothesizes that these widely distributed *rut* sites (i.e., unstructured C-rich sequences) may be the reason that targeting the single-stranded poly(C) region of bacterial RNA is enough for human skin RNases to succeed against bacterial invasion. Once the skin RNase cleaves the *rut* site in a nascent bacterial RNA, the Rho protein can no longer bind to the RNA, and thus will not interact with the RNA polymerase to properly terminate transcription. The author believes she is the first to present the hypothesis that the targeting specificity of human skin RNases may be due to the widely-distributed unstructured C-rich motifs in bacteria, since no related publications have been found to date.

The author proposes that human skin RNases provide dual protection against invading bacteria: They may bind to and disrupt bacterial membranes, and/or may enter bacteria and disrupt their transcription process (see Figure 1). Human skin RNase 7 may be an important defense mechanism against microorganisms that do not use Rho-dependent transcriptional terminators (i.e., their RNAs may not have an unstructured C-rich region), as it can rapidly inactivate these organisms through membrane disruption. For microorganisms that do use Rho-dependent transcriptional terminators, the mechanism(s) by which RNases on human skin gain entrance into the bacterial cell to exert their ribonuclease activity is currently unknown. The author hypothesizes that the RNases may bind to a specific lipoprotein associated with the bacterial membrane leading to the processing and internalization of the membrane protein along with the skin RNases.

**Figure 1:**
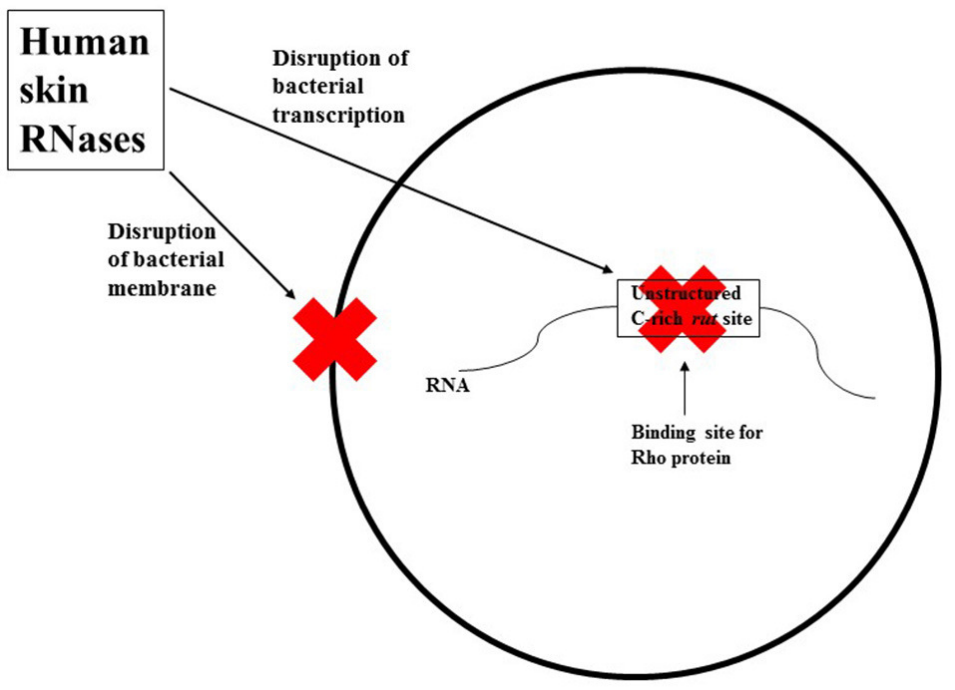
**A simplified schematic representation of the two mechanisms human skin RNases may employ against invading bacteria**. These RNases may bind to and disrupt bacterial membranes, and/or may enter the bacteria and disrupt their transcription process by cleaving the *rut* site (unstructured C-rich sequences) in nascent bacterial RNA.

It is known that several members of both the RNase A superfamily (RNases 1, 4, 5, and 7) and the desquamin class of RNases are expressed in human skin, and that human skin RNases as a whole specifically cleave RNA substrates at single-stranded C-rich regions (Probst et al., 2006; Zasloff, 2009). Among these RNases, RNases 5 and 7 (especially RNase 7) have demonstrated their ability against a variety of microorganisms (Hooper et al., 2003; Pulido et al., 2013; Rademacher et al., 2016,2017). Useful insights on the mechanisms of cutaneous defenses against invading bacteria would be gained from studies focused on the determination of the ribonucleolytic activity of each of the above-mentioned RNases individually. In addition, a comparison of their relative activity would allow the identification of the major contributor to the cleavage of bacterial RNA found on human skin. Furthermore, there may be other proteins on the human epidermis that demonstrate a C-cleaving ribonuclease activity. Research to isolate such RNases and determine their function and activity would contribute to a more complete view of the host-defense system of human skin.

## Author contributions

The author confirms being the sole contributor of this work and approved it for publication.

### Conflict of interest statement

The author declares that the research was conducted in the absence of any commercial or financial relationships that could be construed as a potential conflict of interest.
